# Long Non-Coding RNA MCM3AP-AS1: A Crucial Role in Human Malignancies

**DOI:** 10.3389/pore.2022.1610194

**Published:** 2022-06-16

**Authors:** Tao Ma, Fa-Hong Wu, Hong-Xia Wu, Qiong Fa, Yan Chen

**Affiliations:** ^1^ Department of Hematology, The Affiliated Hospital of Southwest Medical University, Luzhou, China; ^2^ Department of General Surgery Hepatic-Biliary-Pancreatic Institute, Lanzhou University Second Hospital, Lanzhou, China; ^3^ Department of Nuclear Medicine, Lanzhou University Second Hospital, Lanzhou, China; ^4^ Department of Nuclear Medicine, The 940th Hospital of the People’s Liberation Army Joint Service Support Force, Lanzhou, China

**Keywords:** cancer, biomarker, lncRNA, MCM3AP-AS1, clinical features

## Abstract

The incidence of cancer continues to grow and is one of the leading causes of death in the world. Long noncoding RNAs (LncRNAs) is a group of RNA transcripts greater than 200 nucleotides in length, and although it cannot encode proteins, it can regulate different biological functions by controlling gene expression, transcription factors, etc. LncRNA micro-chromosome maintenance protein 3-associated protein antisense RNA 1 (MCM3AP-AS1) is involved in RNA processing and cell cycle-related functions, and MCM3AP-AS1 is dysregulated in expression in various types of cancers. This biomarker is involved in many processes related to carcinogens, such as cell proliferation, apoptosis, cell cycle, and migration. In this review, we summarize the roles of MCM3AP-AS1 in different human cancers and its biological functions with a view to providing ideas for future research.

## Introduction

Cancer is the leading cause of death in the world. Each year, cancer incidence and mortality rates are increasing, with approximately 14.1 million new cases and 8.2 million deaths worldwide in 2012, while this number rose to 18.1 million new cases and 9.6 million deaths in 2018 [1, 2]. The highest incidence of human cancer is lung cancer (LC), followed by female breast cancer (BC), prostate cancer (PCA), and colorectal cancer (CRC), and the highest mortality rate of human cancer is LC, CRC, gastric cancer (GC), and hepatocellular carcinoma (HCC)[2]. Recently, the causes of cancer have been classified as hereditary, infectious and sporadic, with about 5%–10% of cancers thought to be hereditary, about 15% infectious and the rest (70%–80%) classified as sporadic, which is a euphemism for “unknown cause” [3]. There are many treatment options for cancer, including surgery, chemotherapy, radiotherapy, targeted drug therapy, etc., but the prognosis for many cancers is still very poor [4]. More needs to be done to achieve early diagnosis of cancer, targeted therapy, and improved patient survival.

Genomic instability leads to heterogeneity of cancers, so that even the same cancer may have a different prognosis [5]. Transcriptomic analysis shows that up to 85% of genes in the human genome are transcribed, while only 0.1% of genes in the Encyclopedia of DNA Elements (ENCODE) work show mass spectrometric evidence consistent with protein expression, indicating that the majority of human genes are non-coding [6]. Non-coding RNA can be classified into short RNAs and LncRNAs by the size of nucleotides, short RNAs are less than 200 nucleotides, including microRNAs (miRNAs), small interfering RNAs (siRNAs),and piwi-interacting RNAs (piRNAs), while lncRNAs are longer than 200 nucleotides [7]. Present in higher eukaryotes, lncRNAs can form complex secondary structures that regulate the expression of target genes by interacting with DNA-binding proteins, and some lncRNAs can act on adjacent cells *via* exosomes [8–10]. LncRNAs have been shown to be involved in the regulation of a variety of processes, such as hormone receptors, epigenetics, gene expression, transcription factors, and chromosome remodeling [8, 10]. LncRNAs are widely involved in cancer pathogenesis and regulate many cancer-related signalling pathways, such as the p53 pathway and NF-κB pathway [4, 11]. LncRNAs can also promote or inhibit tumour cell proliferation, migration, invasion, apoptosis, epithelial-to-mesenchymal transition (EMT), anti-tumor drug resistance, and affect tumour metastasis [3, 12–16].

MCM3AP-AS1 is localized at chromosome 21q22.3. External Ids for MCM3AP-AS1 gene are HGNC: 16417; NCBI Entrez Gene: 114044; Ensembl: ENSG00000215424. The previous HGNC symbols for the MCM3AP-AS1 gene were C21orf85, MCM3APAS and MCM3AP-AS. Reymond A et al. found C21orf85 when they identified the gene on human chromosome 21 and found that C21orf85 is conserved from rodents to primates [17]. It can be detected in almost all human tissues and organs, including peripheral blood, liver, heart, etc. The functions of MCM3AP-AS1 are diverse and are mainly divided into those in neoplastic diseases and those in non-neoplastic diseases. In non-neoplastic diseases, MCM3AP-AS1 promotes chondrocyte apoptosis, promotes the progression of hemangiomas, regulates the proliferation of human bronchial smooth muscle cells, promotes osteogenic differentiation of dental pulp stem cells, and is associated with acute stroke and coronary artery disease [18–23]. In most tumors, MCM3AP-AS1 mainly shows pro-cancer effects, such as promoting tumor cell proliferation, inhibiting apoptosis and promoting cell metastasis. In most tumors, MCM3AP-AS1 mainly exhibited pro-cancer effects, such as promoting tumor cell proliferation, inhibiting apoptosis and promoting cell metastasis, and in a small number of tumors MCM3AP-AS1 exhibited tumor suppressive effects. This also suggests that the function of MCM3AP-AS1 is two-sided in different tumors. For example, MCM3AP-AS1 is involved in RNA processing and cell cycle-related functions. In lower grade glioma (LGG) (grade II and III), the expression of MCM3AP-AS1 was significantly higher than that of glioblastoma multiforme (GBM), and its expression was significantly downregulated with increasing glioma tumor grade, suggesting that MCM3AP-AS1 may be a protective factor in glioma.[24, 25]. MCM3AP-AS1 is also a hub in prognostic model and lncRNA-associated competing endogenous RNAs (ceRNA) networks in HCC [26].

In this review, we summarize the roles of MCM3AP-AS1 in different human cancers, including expression, targets and signaling pathways, clinical features, biological functions, etc (Table 1).

**TABLE 1:** Role of MCM3AP-AS1 in cancers and its mechanism of action.

Cancers	Expression (tumor vs. normal)	Targets and signaling pathways	Clinical features	Biological functions	References
Hepatocellular carcinoma	Upregulated	miR-194-5p/FOXA1	Positively correlated with large tumour size, high tumour grade, advanced tumour stage and poor prognosis	Knockdown: ↓cell proliferation, colony formation, and cell cycle progression,↑ apoptosis	[27]
	Upregulated	miR-455	Lower overall survival rate	Knockdown:↓metastasis	[28]
Prostate cancer	Upregulated	miR-876-5p/WNT5A	Poor disease free survival	Knockdown: ↓cell proliferation, ↑ apoptosis	[29]
	Upregulated	DNMT1/DNMT3 (A/B) methylation	—	Overexpression: ↑ proliferation, invasion, and migration, ↓ apoptosis	[30]
	Upregulated	miR-543-3p/SLC39A10/PTEN	Knockdown: reduce tumor volume	Overexpression: ↑ proliferation, migration and invasion	[31]
Cervical squamous cell carcinoma	Downregulated	miRNA-93	Poor survival	Overexpression: ↓cell proliferation	[32]
Breast cancer	Upregulated	miR-28-5p/CENPF	—	Knockdown: ↓ cell proliferation, migration and invasion	[33]
Triple negative breast cancer	Upregulated	MEG3	The expression levels of MCM3AP-AS1 increased with the increase in tumor size	Overexpression: ↑cell proliferation	[34]
Colorectal cancer	Upregulated	miR-545/CDK4	Lower overall survival rate	↑ cell proliferation	[35]
	Upregulated	miR-193a-5p/SENP1	Adverse outcomes	Knockdown: ↓ proliferation, colony formation, migration, and invasion	[36]
	Upregulated	miR-599/ARPP19	—	Knockdown: ↓ proliferation and migration	[37]
Papillary thyroid cancer	Upregulated	miR-211-5p/SPARC	Lower long-term survival rate	Knockdown: ↓ proliferation, colony formation, migration and invasion	[38]
Burkitt lymphoma	Upregulated	miR-15a/EIF4E	Poor prognosis	Knockdown: ↓ cell cycle progression, ↑ apoptosis rates	[39]
Pancreatic cancer	Upregulated	miR-138-5p/FOXK1	Shorter survival rates	↑ proliferation, migration, clone formation ability and invasion	[40]
Gastric cancer	Upregulated	miR-708-5p	—	Knockdown: ↓ cell proliferation, ↑ apoptosis	[41]
	Upregulated	miR-138/FOXC1	—	knockdown: ↓ cisplatin resistance	[42]
Lung cancer	Upregulated	miR-340-5p/KPNA4	—	Knockdown: ↓ cell proliferation, migration, and angiogenesis	[43]
Clear cell renal cell carcinoma	Upregulated	DPP4	Poor recurrence-free survival and overall survival	↑ cell proliferation, ↑ the release of pro-inflammatory cytokines	[44]
oral squamous cell carcinoma	Upregulated	miR-204-5p/FOXC1	—	↑ proliferation, migration and invasion	[45]
	Upregulated	miR-363-5p	poor prognosis	↑ proliferation, migration and invasion	[46]
Nasopharyngeal Carcinoma	Upregulated	miR-34a	poor survival	Knockdown: ↓ cell proliferation, ↑ apoptosis	[47]

## MCM3AP-AS1 in Human Tumors

### Hepatocellular Carcinoma

Liver cancer is a significant cause of cancer-related deaths worldwide, with more than 850,000 cases worldwide each year, and HCC accounts for approximately 90% of all primary liver cancers [2, 48]. MCM3AP-AS1 is a hub in prognostic model and lncRNA-associated ceRNA networks in HCC [26]. Although MCM3AP-AS1 is a protective gene in glioma, MCM3AP-AS1 has a pro-oncogenic effect in HCC, and its function may be influenced by the pathogenesis, site, and tumor cell characteristics of the tumor. The specific reasons for this discrepancy need further investigation. One study found that knockdown of MCM3AP-AS1 can reduce invasion of HCC cells and lymphatic vessel formation capabilities, MCM3AP-AS1 interacted directly with miR-455, and inhibitors of miR-455 enhanced invasion of HCC cells and lymphatic vessel formation capabilities [28]. Mimics of miR-455 inhibited the invasion of HCC cells and lymphatic vessel formation capabilities, which were counteracted by the overexpression of the autophagy- related gene ATG7 [28]. This study also found that patients with HCC who had low levels of MCM3AP-AS1 had better survival rates [28]. This study focused on MCM3AP-AS1 and the invasion of HCC cells and lymphatic vessel formation capabilities, and the relevance of MCM3AP-AS1 to the clinic was less involved. And another study remedied the information. MCM3AP-AS1 was found to be overexpressed in HCC and positively correlated with poor prognosis, large tumor size, and advanced tumor stage, high tumor grade in HCC patients [27]. Knockdown MCM3AP-AS1 inhibited proliferation, cell cycle progression, colony formation, and induction of apoptosis in HCC cells [27]. Further studies revealed that MCM3AP-AS1 promotes HCC cell growth by targeting the miR-194-5p/forkhead box A1 (FOXA1) axis, MCM3AP-AS1 promoted FOXA1 gene expression and FOXA1 restoration rescued MCM3AP-AS1 knockdown induced proliferation inhibition, G1 blockade and apoptosis in HCC cells [27]. These studies suggest that overexpression of MCM3AP-AS1 is associated with poor prognosis of HCC, which is acting through different pathways, that MCM3AP-AS1 may serve as a target for HCC therapy, and that the synergistic effect of MCM3AP-AS1 knockdown with therapeutic drugs for HCC could be a direction for further research.

### Prostate Cancer

The most common non-cutaneous cancer in men worldwide is PCA, although the prognosis of PCA has improved in recent years, it remains a major cause to cancer-related deaths in men [49, 50]. To further improve the therapeutic effects of PCA, more relevant studies are needed. It was found that MCM3AP-AS1 is an important target for PCA metastasis and improve poor prognosis, and valproic acid and trichostatin A may be potential therapeutic agents for PCA by reversing the expression level of MCM3AP-AS1 [51]. MCM3AP-AS1 was upregulated in PCA, and overexpression of MCM3AP-AS1 promoted PCA cell proliferation, invasion and migration, while reducing PCA cell apoptosis[29–31]. Patients with high expression of MCM3AP-AS1 had worse disease-free survival, while knockdown of MCM3AP-AS1 in animal experiments reduced tumor volume in PCA [29, 31]. MCM3AP-AS1 can function in PCA through multiple pathways, such as miR-876-5p/WNT5A axis, DNMT1/DNMT3 (A/B) methylation and miR-543-3p/SLC39A10/PTEN axis [29–31]. More studies have focused on the biological role and mechanism of action of MCM3AP-AS1 in PCA, but there is a lack of data on its correlation with the clinical characteristics of PCA patients and there is no research on its status in the treatment of PCA, which is a future research direction.

### Cervical Squamous Cell Carcinoma

Cervical cancer is the fourth most common malignancy among women worldwide, ranking fourth in both incidence and mortality [2]. In 2018, 569,847 new cases of cervical cancer were diagnosed, accounting for 3.2% of all new cancer cases, and 311,365 cervical cancer deaths, accounting for 3.3% of all cancer deaths [2]. LncRNAs played an important role in the prognosis and tumor progression, invasion, metastasis, and apoptosis of cervical cancer [52]. The role of MCM3AP-AS1 in CSCC was investigated by Lan L et al [32]. They studied specimens from 64 CSCC patients and two human CSCC cell lines and found that MCM3AP-AS1 expression was downregulated in CSCC patients and the lower the expression of MCM3AP-AS1, the lower the overall survival rate of the patients [32]. MCM3AP-AS1 inhibits CSCC cell proliferation by negatively regulating miR-93 [32]. In CSCC, MCM3AP-AS1 expression is downregulated, which is contrary to the expression of MCM3AP-AS1 in some other tumors [30–32]. We need to conduct more studies to determine which downstream pathways MCM3AP-AS1 can affect in CSCC and whether MCM3AP-AS1 has any effect on apoptosis, cell cycle, drug resistance, metastasis, etc. in CSCC cells.

### Breast Cancer

BC is the most common cancer in women [2, 53]. BC is also one of the three most common cancers in humans, the other two being LC and PCA. In 2018, there were 2,088,849 new diagnoses of BC, accounting for 11.6% of all new cancer cases, and 626,679 deaths from BC, accounting for 6.6% of all cancer deaths [2]. MCM3AP-AS1 expression was higher in BC tissues than in paracancerous tissues, and it was mainly distributed in the cytoplasm of BC cell lines (MCF-7 and BT-549) [33, 54]. Overexpression of CENPF in BC tissues and cells contributed to cell proliferation, migration and invasion of BC [33]. Overexpression of miR-28-5p inhibited the protein level of CENPF while MCM3AP-AS1 decreased the expression of miR-28-5p, so overexpression of MCM3AP-AS1 increased the expression of CENPF [33]. *In vivo* experiments also confirmed that MCM3AP-AS1 regulates cellular processes and accelerates tumor growth through the miR-28-5p/CENPF axis in BC [33]. In triple-negative breast cancer (TNBC), the expression level of MCM3AP-AS1 increased with tumor size, and overexpression of MCM3AP-AS1 led to an increased proliferation rate of tumor cells through downregulation of MEG3 [34]. here are still relatively few studies focusing on the role of MCM3AP-AS1 in BC, mainly on basic research, and future research combining basic and clinical studies is needed.

### Colorectal Cancer

CRC is the second most common cancer in women and the third most common cancer in men, and it accounts for approximately 10% of diagnosed cancer and cancer-related deaths worldwide each year [55]. The main methods of treating CRC are surgery, targeted therapy, and chemotherapy, and the efficacy of treatment has improved compared with the past, but some patients still have problems such as recurrence and metastasis leading to a poor prognosis, and understanding the mechanism of CRC progression is important to improve the prognosis of CRC [56]. Ma X et al. used RT-qPCR to detect the expression level of MCM3AP-AS1 in CRC and paraneoplastic tissues, and found that the expression level of MCM3AP-AS1 was significantly higher in CRC tissues, and patients in the high MCM3AP-AS1 level group [35] had a lower overall survival rate compared with those in the low MCM3AP-AS1 level group, but the expression level of MCM3AP-AS1 did not correlate with patients’ age, gender, tumor stage, or tumor grade [35]. MCM3AP-AS1 and miR-545 interacted but did not regulate each other’s expression. Overexpression of MCM3AP-AS1 increased the expression level of CDK4 and promoted CRC cell proliferation, while overexpression of miR-545 decreased the expression level of CDK4. Overexpression of MCM3AP-AS1 and CDK4 decreased the percentage of G1-phase cells and increased the percentage of G2-phase cells. Nude mice transplantation tumor experiments revealed that the mean tumor volume and weight of CRC cells overexpressing MCM3AP-AS1 increased compared to the control group, and detection of ki-67 in tumor tissues suggested increased proliferation of tumor tissues in the MCM3AP-AS1 overexpression group [35]. Another study also found that MCM3AP-AS1 expression was upregulated in CRC and predicted poor clinical prognosis [36]. MCM3AP-AS1 not only promoted CRC cell proliferation but also its metastasis, colony formation, migration, and invasive, and MCM3AP-AS1 played an oncogenic role in CRC through the miR-193a-5p/SENP1 axis [36] Yu Y et al. suggested that MCM3AP-AS1 promotes CRC cell proliferation and metastasis through the miR-599/ARPP19 axis, ultimately accelerating CRC progression [37]. Several studies have confirmed that MCM3AP-AS1 promotes proliferation and metastasis of CRC cells, and that MCM3AP-AS1 is associated with a poorer prognosis in CRC patients, and that MCM3AP-AS1 has multiple downstream pathways to achieve this effect [35–37]. The effect of MCM3AP-AS1 on the progression of CRC cells is obvious, and more studies could target whether MCM3AP-AS1 is associated with drug resistance and angiogenesis in CRC cells.

### Papillary Thyroid Cancer

PTC is the most common type of endocrine malignancy and the most common type of thyroid cancer, accounting for 3.4% of all new tumors [57]. Surgery and radiation therapy are effective ways to treat PTC and could cure most patients, but about 10% of cases can still differentiate into the more aggressive and lethal thyroid cancer [58]. A better understanding of the pathogenesis of PTC will help us to improve its treatment. Comparison of tumor tissues with paraneoplastic tissues from 68 PTC patients revealed that MCM3AP-AS1 expression was upregulated in PTC, and the long-term survival rate of PTC with high MCM3AP-AS1 expression was significantly lower than that of the low expression group [38]. In several cell lines of PTC, the expression of MCM3AP-AS1 was also upregulated compared to normal thyroid epithelial cells, and inhibition of MCM3AP-AS1 expression inhibited proliferation, colony formation, migration and invasion of PTC cells [38]. *In vivo* studies have shown that mice inoculated with PTC cells overexpressing MCM3AP-AS1 have reduced tumor volume compared to controls [38]. MCM3AP-AS1 effect on PTC is mainly achieved through the miR-211-5p/SPARC axis. The current study on MCM3AP-AS1 and thyroid cancer is only limited to PTC, and further studies can be done on other types of thyroid cancer in the future.

### Burkitt Lymphoma

BL is an aggressive, rare non-Hodgkin’s lymphoma that is curable in children and young adults and has a poor prognosis in middle-aged and elderly populations, with many patients experience chemotherapy resistance and develop refractory disease [39, 59]. LncRNAs affect the pathogenesis and prognosis of many lymphomas [60–62]. MCM3AP-AS1 expression was upregulated in BL compared to normal lymph nodes, and age and gender factors were not associated with MCM3AP-AS1 expression levels, tumor size and tumor stage were positively associated with MCM3AP-AS1 expression levels, and patients with low MCM3AP-AS1 expression had a better long-term prognosis than patients with high MCM3AP-AS1 expression [39]. In BL, knockdown of MCM3AP-AS1 can enhance drug sensitivity, promote cell cycle progression, and promote apoptosis by regulating EIF4E and its downstream anti-apoptotic proteins [39]. MiR-15a as a connecter between MCM3AP-AS1 and EIF4E, and the MCM3AP-AS1/miR-15a/EIF4E axis may be a promising target for the treatment of BL [39]. The high expression of MCM3AP-AS1 in BL is a poor prognostic factor, and the role of MCM3AP-AS1 in other lymphomas deserves to be further investigated.

### Pancreatic Cancer

PC is a fatal disease that is easily missed in its early stages, and <5% of PC patients are still alive after 5 years [63]. LncRNAs can be used as markers for early diagnosis of PC, prognostic biomarkers, and new therapeutic approaches [64]. It was found that MCM3AP-AS1expression was upregulated in PC tissues compared to paraneoplastic tissues and that patients with higher levels of MCM3AP-AS1 expression had shorter survival rates. MCM3AP-AS1 expression was also up-regulated in PC cell lines [40]. MCM3AP-AS1 overexpression in PC cell lines promoted PC cell proliferation, clone formation ability, invasion and migration ability [40]. Knockdown of MCM3AP-AS1 in PC cell lines inhibited PC cell proliferation and invasive ability, and *in vivo* experiments showed that knockdown of MCM3AP-AS1 inhibited tumor growth in mice, including a reduction in tumor volume and weight [40]. MCM3AP-AS1 negatively associated with miR-138-5p expression, and MCM3AP-AS1 promoted the expression of FOXK1 through miR-138-5p, thus promoted the growth and invasion of PC cells [40]. Fewer studies have been conducted on the relationship between MCM3AP-AS1 and PC, and more studies can focus on whether there is a correlation between MCM3AP-AS1 and clinical features of PC and the relationship between MCM3AP-AS1 and PC treatment.

### Gastric Cancer

GC has the fifth highest incidence rate and the third highest mortality rate of all cancers. Despite recent advances in the diagnosis and treatment of GC, many patients are often at an advanced stage at the time of diagnosis, and long-term survival rates for GC are rather low [65]. LncRNAs play a key role in the development of GC and GC resistance to chemotherapeutic agents and targeted therapeutics [66]. One study found that MCM3AP-AS1 expression was upregulated in cisplatin-resistant GC cells, and MCM3AP-AS1 enhanced cisplatin resistance in GC cells by upregulating FOXC1 expression through spongy miR-138 [42]. MCM3AP-AS1 also had an important role in the regulation of proliferation and apoptosis of GC cells. The expression of MCM3AP-AS1 was found to be upregulated in GC cell lines using qRT-PCR assay, and knockdown of it significantly inhibited GC cell proliferation and promoted apoptosis [41]. MiR-708-5p is a target gene of MCM3AP-AS1, and knockdown of miR-708-5p rescued the effect of MCM3AP-AS1 on GC cell proliferation and apoptosis [41]. MCM3AP-AS1 has an effect on GC not only in cell proliferation and apoptosis but also in GC cell resistance, and the role of MCM3AP-AS1 in GC needs to be further investigated.

### Lung Cancer

LC is the cancer with the highest number of newly diagnosed patients and cancer-related deaths in the world each year [2]. Its early diagnosis is difficult, effective treatment strategies are lacking, and 5-year survival rates remain low [67]. LncRNAs play an important role in cell proliferation, apoptosis, and metastasis in LC [67]. The investigators used RT-qPCR to assess the expression of MCM3AP-AS1 in LC cells and found that MCM3AP-AS1 expression was significantly upregulated, and knockdown of MCM3AP-AS1 inhibited angiogenesis and progression of LC [43]. The results of Chromatin Immunoprecipitation (ChIP) assay showed that YY1 could bind to the MCM3AP-AS1 promoter, and the upregulation of YY1 significantly enhanced the expression of MCM3AP-AS1. MCM3AP-AS1 sponged miR-340-5p in LC cells, and KPNA4 was a downstream target of miR-340-5p [43]. Overexpression of KPNA4 counteracted the inhibitory effect of knockdown MCM3AP-AS1 on LC cell proliferation and restored the expression of angiogenesis-related proteins [43]. MCM3AP-AS1 can accelerate angiogenesis and progression of LC by targeting the miR-340-5p/KPNA4 axis [43]. The effect of MCM3AP-AS1 on LC angiogenesis and progression has been studied, but additional studies combined with clinical data are needed to determine the role of MCM3AP-AS1 in LC pathogenesis, disease progression, treatment and prognosis.

### Clear Cell Renal Cell Carcinoma

The average age of diagnosis of renal cell carcinoma is about 60 years old, with men accounting for two-thirds of the cases [68]. ccRCC accounts for about 70% of renal cell carcinoma, and most ccRCC can be detected early and treated successfully by surgery and other ways, but about one-third of patients still develop metastasis, and the prognosis of patients after metastasis is extremely poor [68]. In ccRCC, MCM3AP-AS1 was the most differentially expressed lncRNA from normal samples [44]. The expression of MCM3AP-AS1 in paraneoplastic tissues and ccRCC tissues was assayed by RT-qPCR, and it was found that the expression of MCM3AP-AS1 was upregulated in ccRCC tissues, and the higher the grade of ccRCC tissues, the upregulation was more obvious, and the expression of MCM3AP-AS1 in ccRCC tumors >7 cm was significantly higher than that in tumors ≤7 cm [44]. Patients with lower MCM3AP-AS1 expression had higher recurrence-free survival and overall survival relative to those with higher MCM3AP-AS1 expression, and high MCM3AP-AS1 expression was an independent prognostic factor for ccRCC patients [44]. In both *in vivo* and *in vitro* experiments, downregulation of MCM3AP-AS1 was found to inhibit tumorigenicity, tumor-associated inflammation and angiogenesis in ccRCC, and MCM3AP-AS1 upregulated the DPP4 gene by entrapment E2F1 into the DPP4 gene promoter to achieve regulation of pro-angiogenesis and pro-inflammation in ccRCC [44]. Studies have shown that MCM3AP-AS1 was associated with prognosis and tumor aggressiveness of ccRCC, and more studies can be done on drug resistance in the future.

### Oral Squamous Cell Carcinoma

OSCC is the most common malignancy in the oral cavity, and despite recent improvements in its diagnosis and treatment modalities, its morbidity and mortality rates are still on the rise, and its 5-year survival rate has not improved significantly in the last decade, ranging from 45%–50% [69, 70]. Many lncRNAs are dysregulated and associated with OSCC and affect various aspects of OSCC cells, including proliferation, survival, migration, or genomic stability [70]. One study using qRT-PCR to detect MCM3AP-AS1 expression in OSCC tissues and cells found that MCM3AP-AS1 expression was elevated in OSCC tissues and cell lines. Overexpression of MCM3AP-AS1 promoted proliferation, migration and invasion of OSCC cells, which was achieved through the inhibition of miR-204-5p expression by MCM3AP-AS1, thereby upregulating FOX1 [45]. Another study also found that MCM3AP-AS1 expression was upregulated in OSCC, and the expression level of MCM3AP-AS1 was significantly correlated with clinical stage and lymph node metastasis, with a higher percentage of high MCM3AP-AS1 expression in patients with stage III/IV and positive lymph node metastasis [46]. Knockdown of MCM3AP-AS1 inhibited the proliferation, migration and invasion of OSCC cells. MCM3AP-AS1 directly inhibited the expression of miR-363-5p in OSCC cells, and downregulation of miR-363-5p reversed the effects of knockdown MCM3AP-AS1 on proliferation, migration and invasion of OSCC cells [46]. MCM3AP-AS1 can promote the proliferation, migration and invasion of OSCC cells through multiple pathways, and MCM3AP-AS1 is an important target for the development of OSCC.

### Nasopharyngeal Carcinoma

In 2018, there were 129,079 new cases of NPC, accounting for 0.7% of all new cancers, and 72,987 deaths, accounting for 0.8% of all cancer-related deaths [2]. Patients with early diagnosis of stage I NPC have a good prognosis and even achieve cure, but less than 10% of patients can be diagnosed early, and once metastasis occurs, the 5-year overall survival rate of NPC drops to less than 40% [47]. Sun P et al. studied the expression levels of MCM3AP-AS1 and miR-34a in NPC tissues and paracancerous tissues of 55 NPC patients, and found that the expression levels of miR-34a were significantly lower and those of MCM3AP-AS1 were significantly higher in NPC tissues compared with paracancerous tissues, and the expression of both was negatively correlated. The overall survival rate of NPC patients with high expression of MCM3AP-AS1 was lower. MCM3AP-AS1 overexpression promoted cell proliferation and inhibited apoptosis, while overexpression of miR-34a resulted in downregulation of MCM3AP-AS1 expression [47]. It was demonstrated that MCM3AP-AS1 was overexpressed in NPC and promoted NPC cell proliferation and tumor proliferation, and miR-34a could inhibit the above effects [47]. The relationship between A and NPC needs to be further demonstrated by more clinical studies combined with *in vitro* and *in vivo* studies.

## Conclusion

**FIGURE 1:**
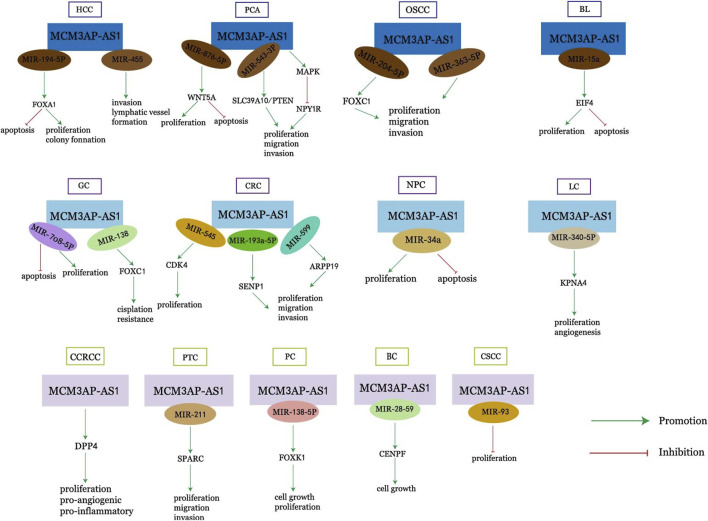
Mechanism of action of MCM3AP-AS1 in cancers.

LncRNAs have important roles in cancer development, and MCM3AP-AS1, a novel LncRNA, is upregulated in most cancers. It can affect a variety of biological properties of cancer, including cell proliferation, apoptosis, migration, cell cycle, and angiogenesis, and is associated with a variety of clinical characteristics of cancer, such as overall survival, disease-free survival, tumor volume, tumor stage, tumor drug resistance, and tumor prognosis. MCM3AP-AS1 can exert its effects through various pathways such as miR-194-5p/FOXA and miR-138-5p/FOXK1 (Figure 1). In conclusion, MCM3AP-AS1 can serve as a new diagnostic or prognostic marker for cancer and is expected to be a promising therapeutic target for cancer. In most cancers, the role and mechanism of action of MCM3AP-AS1 has been largely clarified, but it has not been studied in leukemia, multiple myeloma, gallbladder cancer, bladder cancer, esophageal cancer, and other cancers, and these are also directions for future research. RNA-targeted therapeutics include oligonucleotides, mRNA and RNA-associated small molecules. Four RNA-targeted therapies have been approved for commercial use, and key approaches to address delivery challenges - including chemical modification, bioconjugation and the use of nanocarriers - are also being investigated. Oligonucleotides are a potential strategy for drug research and development [71, 72]. Targeted therapy against MCM3AP-AS1 is also one of the future research directions for tumor treatment.
